# Probing the Importance of Charge Balance and Noise Current in WSe_2_/WS_2_/MoS_2_ van der Waals Heterojunction Phototransistors by Selective Electrostatic Doping

**DOI:** 10.1002/advs.202001475

**Published:** 2020-08-18

**Authors:** Hyun‐Soo Ra, Min‐Hye Jeong, Taegeun Yoon, Seungsoo Kim, Young Jae Song, Jong‐Soo Lee

**Affiliations:** ^1^ Department of Energy Science and Engineering Daegu Gyeongbuk Institute of Science and Technology (DGIST) Daegu 42988 Republic of Korea; ^2^ Department of Nano Engineering Sungkyunkwan University (SKKU) Suwon 16419 Korea; ^3^ SKKU Advanced Institute of Nano Technology (SAINT) Sungkyunkwan University (SKKU) Suwon 16419 Korea

**Keywords:** electrostatic doping, heterojunctions, noise current, scanning photocurrent mapping, transition metal dichalcogenides

## Abstract

Heterojunction structures using 2D materials are promising building blocks for electronic and optoelectronic devices. The limitations of conventional silicon photodetectors and energy devices are able to be overcome by exploiting quantum tunneling and adjusting charge balance in 2D p–n and n–n junctions. Enhanced photoresponsivity in 2D heterojunction devices can be obtained with WSe_2_ and BP as p‐type semiconductors and MoS_2_ and WS_2_ as n‐type semiconductors. In this study, the relationship between photocurrent and the charge balance of electrons and holes in van der Waals heterojunctions is investigated. To observe this phenomenon, a p‐WSe_2_/n‐WS_2_/n‐MoS_2_ heterojunction device with both p–n and n–n junctions is fabricated. The device can modulate the charge carrier balance between heterojunction layers to generate photocurrent upon illumination by selectively applying electrostatic doping to a specific layer. Using photocurrent mapping, the operating transition zones for the device is demonstrated, allowing to accurately identify the locations where photocurrent generates. Finally, the origins of flicker and shot noise at the different semiconductor interfaces are analyzed to understand their effect on the photoresponsivity and detectivity of unit active area (2.5 µm^2^, *λ* = 405 nm) in the p‐WSe_2_/n‐WS_2_/n‐MoS_2_ heterojunction device.

## Graphical abstract

By selectively applying electrostatic doping to a specific channel layer, a p‐WSe_2_/n‐WS_2_/n‐MoS_2_ heterojunction device with both p–n and n–n junctions can modulate the charge carrier balance between heterojunction layers to generate photocurrent upon illumination. The relationship between photocurrent and the charge balance of electrons and holes in van der Waals heterojunctions is investigated.

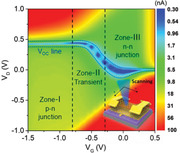

## 1 Introduction

2D transition metal dichalcogenides (TMDC) have attracted great attention as ideal building blocks for electronic and optoelectronic applications.^[^ ^1^, ^2^, ^3^, ^4^, ^5^, ^6^, ^7^, ^8^ ^]^ Depending on their thickness, TMDCs show excellent charge mobility due to 2D electron gas (2DEG) formation on the *x*–*y* plane and surface charge screening effect resulting from the 2D material structure.^[^ ^9^, ^10^, ^11^ ^]^ Furthermore, single‐layer TMDCs have a direct bandgap and possess excellent absorption characteristics.^[^ ^12^, ^13^ ^]^ To achieve high‐efficiency TMDC‐based optoelectronic devices, the photocurrent generation and charge balance must be optimized. High responsivity and high detectivity TMDC phototransistors require ideal charge depletion conditions to produce low dark currents. However, photocurrent generation in TMDCs is limited because there is not enough energy to split the excitons due to their high exciton binding energy (≈0.897 eV).^[^ ^12^, ^14^, ^15^ ^]^ To solve such inherent challenges, a method for forming a built‐in potential (*V*_bi_) by the creation of a heterojunction structure in the device channel was introduced.^[^ ^5^, ^16^, ^17^, ^18^, ^19^, ^20^, ^21^ ^]^ Heterojunction structures typically consist of junctions of different n‐type and p‐type semiconductors.^[^ ^5^, ^16^, ^17^, ^18^, ^19^, ^20^, ^21^ ^]^ For optimal photocurrent generation in a van der Waals heterojunction, forming an optimal *V*_bi_ in the device is crucial.^[^ ^5^, ^17^, ^22^ ^]^ The *V*_bi_ can be created by controlling the balance of electron and hole concentrations and the proper doping of 2D materials. However, chemical doping of the 2D materials remains difficult because of self‐purification in low‐dimensional systems.^[^ ^23^, ^24^ ^]^ Also, it is important to systematically determine the origins of photocurrent and noise in van der Waals heterojunction devices to maximize the critical photodetector figure of merit such as responsivity (A W^−1^), and detectivity (D*, cm Hz^1/2^ W^−1^). However, the effect of the *V*_bi_ in the van der Waals heterojunction to photocurrent generation efficiency as well as the effect of flick noise and shot noise on the responsivity and detectivity have not yet been elucidated.

To identify the origins of photocurrent generation and noise in van der Waals heterojunctions, we fabricated a multifunctional 2D heterojunction phototransistor with a lateral p‐WSe_2_/n‐WS_2_/n‐MoS_2_ structure. Both p–n and n–n junctions in the heterojunction phototransistor were designed to control the major charge carriers by applying the gate bias to the WS_2_ layer only. We used a thin hexagonal‐BN (h‐BN) layer as a dielectric material to minimize dielectric traps and the operation gate voltage.^[^ ^25^, ^26^ ^]^ In these devices, we analyzed the density of charge carriers precisely and the exact position of photocurrent generation as a function of gate bias using scanning photocurrent mapping system (beam size: 898 nm). We also identified the origins of flicker noise and shot noise according to the charge balance between electrons and holes. When the optimum charge concentration (built‐in potential) was attained, the device exhibited fast time‐resolved photocurrent response (<10 ms), high responsivity (≈10^6^ A W^−1^), and high D* (≈10^15^ cm Hz^1/2^ W^−1^) in a unit of acitve area (2.5 µm^2^, *λ* = 405 nm).

## 2 Results and Discussion

**Figure 1:**
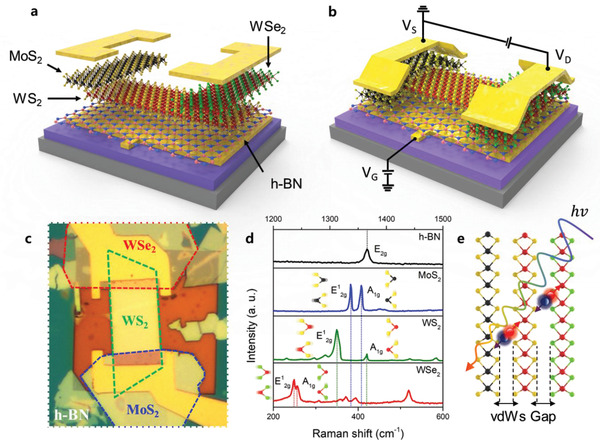
Device design and schematic diagram, and TMDC Raman characteristics. a) Device design and TMDC junction schematic diagram, following this order: MoS_2_–WS_2_–WSe_2_ on h‐BN (index: black ball: molybdenum; red ball: tungsten; yellow ball: sulfide; green ball: selenide). Gate field effect via h‐BN only affect WS_2_ layer. b) Field‐effect phototransistor. Prepattern on SiO_2_ is gate electrode with *V*_G_. Source and drain electrodes were deposited on MoS_2_ and WSe_2_ as n‐type and p‐type layers. c) Optical microscopy image. d) Raman spectroscopy of TMDCs, such as E^1^_2g_ of in‐plane and A_1g_ of out of plane vibration type, E_2g_ of h‐BN. e) Schematic diagram about interface exciton generation and separation mechanism at van der Waals gap of MoS_2_–WS_2_–WSe_2_ heterojunction. Blue–red ball indicates electron and hole.

To control the major charge concentration of the 2D materials, we designed a p–n–n heterojunction device with cascade band alignment and applied the gate bias on only the WS_2_ layer. The p–n–n device demonstrates the origin of photocurrent generation and noise for the different junction types (p–n and n–n junctions) by tuning the Fermi level of the intrinsic WS_2_ layer. The p–n–n device is composed of prepatterned electrodes formed on silicon substrate with a 300 nm  thick SiO_2_ dielectric layer applying a global gate voltage, and the main structure formed by stacking thin h‐BN layers as a dielectric on the gate electrode. By using of h‐BN the layered structure is formed with no mutual coupling with the other 2D materials, reducing interface and vacancy trap effects and maximizing gate efficiency (*C*_h‐BN_ = 1.20 × 10^−3^ F m^−2^).^[^ ^27^, ^28^ ^]^ A thin WS_2_ layer exfoliated from bulk flakes is then stacked over the h‐BN dielectric layer. WSe_2_ as p‐type semiconductor and MoS_2_ as n‐type semiconductor are stacked on both ends of the WS_2_ sheet to form the p–n–n junction as shown in Section S1 (Supporting Information). Finally, as shown in **Figure** **1**a, source and drain electrodes were selectively deposited on WSe_2_ and MoS_2_ stacked on WS_2_, respectively, using PMMA‐masked electron beam lithography (EBL). Figure 1b shows the final p–n–n device structure and the phototransistor circuit and an optical image of the fabricated device is provided in Figure 1c.

By observing the in‐plane (E^1^_2g_) and out‐of‐plane (A_1g_) vibration modes for each materials using Raman spectroscopy, we were able to identify the WSe_2_, WS_2_, MoS_2_, and h‐BN layers (Figure 1d).^[^ ^18^, ^26^, ^29^ ^]^ Particularly, even if each TMDC layer forms van der Waals heterojunctions, the vibration energy of each layer does not exhibit any significant change because there is no primary chemical bonding structure on the *z*‐axis and every stacked layer is isolated.^[^ ^30^ ^]^ The van der Waals gap in the *z*‐axis of the 2D materials plays an important role as a charge separation barrier between interlayers. Thus, 2D materials have the advantage of efficiently collecting free carriers based on their excellent charge mobility on the *x*–*y* plane to maximize optoelectronic properties (Figure 1e).^[^ ^31^ ^]^

**Figure 2:**
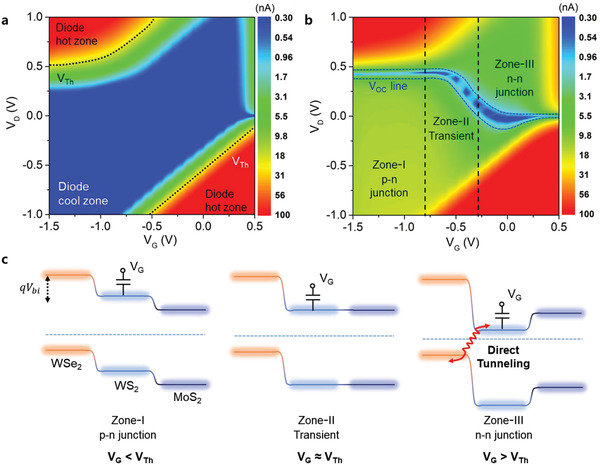
Optoelectronic properties and band alignment of multifunctional 2D heterojunction phototransistor. a) Current mapping as a function of gate bias from −1.5 to 0.5 V_G_ and drain bias from −1 to 1 V_D_. b) Photocurrent mapping under incident light power of 52 µW cm^−2^ at the same bias condition and current range from 0.3 to 100 nA. *V*_OC_ is indicated by blue dashed lines for a guide to the eye. c) Cascade band alignment of WSe_2_–WS_2_–MoS_2_ for explaining the operating mechanism in three zones according to gate bias (capacitance symbol) of WS_2_.

To demonstrate the optoelectronic properties of the p–n–n cascade phototransistor, we first measured the transfer curves for WSe_2_–WS_2_–MoS_2_ devices as a function of gate voltages from −6 to 2 V_G_ after thermal annealing to enhance the p‐type property of WSe_2_ as detailed in Section S2 (Supporting Information).^[^ ^32^, ^33^ ^]^ The device, measured from −1.5 to 0.5 V_G_ region under dark conditions, shows an ideal diode behavior with two different directions as shown in **Figure** **2**a and Section S3 (Supporting Information). In Figure 2a, current mapping shows a typical diode transition at −0.5 V_G_ in the reverse‐cool zone and the forward‐hot zone according to the applied gate voltage and drain voltage. Notably, the threshold voltage (*V*_Th_) as indicated by the black dash line shows two different diode directions due to proportion to the threshold barrier of *qV*_bi_ = *ϕ*_p_−*ϕ*_n_ at the relative p–n junction, where *q* is electron charge and *ϕ* is work function. As a function of the gate bias, shifting the Fermi level of WS_2_ leads to a change in the threshold barriers in the WSe_2_ and MoS_2_ junctions. The bulk bandgap (*E*_g_) of TMDCs is around ≈1.3 eV, and the *V*_bi_ in the junction according to the Fermi level alignment can be estimated using the following p–n junction formula^[^ ^29^, ^34^, ^35^ ^]^

(1) $$\begin{array}{c} qV_{\mathrm{bi}}=\left[E_{g}-KT\left(\ln\frac{N_{C}}{N_{D}^{\prime }}+\ln\frac{N_{V}}{N_{A}^{\prime }}\right)\right] \end{array}$$

where *N*_C_ is the effective concentration of electrons in the conduction band, *N*_V_ is the effective concentration of holes in the valence band, and *N*′_D_ and *N*′_A_ are the net donor and acceptor concentrations, respectively.

Since only the Fermi level of WS_2_ (*N*_C_/*N*′_D_) can be tuned as a function of the gate bias, *N*_V_/*N*′_A_ can be considered as a constant. Therefore, the change in *V*_Th_ depends on the change in *qV*_bi_ according to the applied gate bias to WS_2_. When white light (52 µW cm^−2^) illuminated the device, the photovoltaic effect was observed as shown in Figure 2b and Section S4 (Supporting Information). In the cool zone of the diode in Figure 2a, the net current increased from 10^−12^ to 10^−9^ A by photocurrent generation. In the specific gate range −1.5 to −0.5 V_G_, we observed a typical photovoltaic effect, and it gradually disappeared above the −0.5 V_G_ region. The open‐circuit voltage (*V*_OC_) measured in the range of −0.9 to −0.7 V_G_ is 0.42 V, which is higher than the 0.33 V  observed in the previously reported BP/WS_2_ heterojunction devices.^[^ ^19^ ^]^ The *V*_OC_ as a function of the applied gate bias was changed as marked by the blue line in zero current line. To understand the mechanism for the photovoltaic effect as a function of gate bias in detail, the photocurrent map was divided into three different transition zones: Zone‐I is the p–n junction, where *V*_OC_ is maintained; Zone‐II is a transient region, where *V*_OC_ is shrinking; and Zone‐III involves the n–n junction, where *V*_OC_ has vanished. The photovoltaic effect can be understood by using the band diagram shown in Figure 2c. Since the applied gate field has an effect only on the WS_2_ layer (capacitor symbol), only the WS_2_ layer in the heterojunction band diagram shows a Fermi level shift. We clearly confirmed the Fermi level shift of WS_2_ as a function of the applied gate bias through Kelvin probe force microscope measurement (KPFM) in Section S2 (Supporting Information). The Zone‐I transition (*V*_G_ < *V*_Th_) shows the primary cascade p–n–n band alignment which has a sufficient band offset between the valence band of WSe_2_ and the conduction band of WS_2_ upon illumination with white light (52 µW cm^−2^), exhibiting the photovoltaic effect with *V*_OC_. The Zone‐II transient state (*V*_G_ ∼ *V*_Th_) shows that the band offset of WSe_2_ and WS_2_ are smaller than in Zone‐I and as a result, *V*_OC_ almost disappears. In the Zone‐III transient state (*V*_G_ > *V*_Th_), as the potential barrier between the conduction band of WS_2_ and WSe_2_ narrows further, the transport between WSe_2_ and WS_2_ involves direct tunneling, which is similar to previous reports.^[^ ^22^ ^]^ Therefore, the n–n junction between WS_2_ and MoS_2_ is preferentially created as shown in the Zone‐III transition state in Figure 2c, and the direction of the diode is reversed.

To identify the precise positions of photocurrent generation in the three different zones, the scanning photocurrent mapping with a 405 nm  laser with a divergence of 0.5 mrad was performed as detailed in Section S5 (Supporting Information). The transient zones have a unique output curve where different built‐in barriers are found along the drain voltage direction at −0.3 V_G_ as described in Section S6 (Supporting Information). The position of photocurrent generation varied depending on the transition zone. The photocurrent map was generated by applying −0.5, 0, and 0.5 V_D_ at constant −0.3 V_G_ to investigate the exact positions of photocurrent generation by the device at forward and reverse bias.

**Figure 3:**
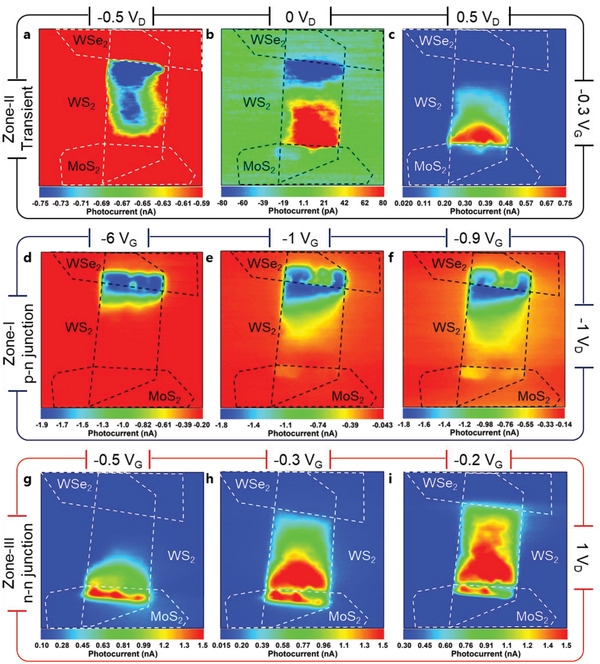
Scanning photocurrent mapping by 405 nm  laser of 20 nW cm^−2^ as a function of gate and drain bias. Material positions of WSe_2_, WS_2_, and MoS_2_ represented as dash lines for a guide to the eye. a–c) Photocurrent measured at −0.5, 0, 0.5 V_D_ under −0.3 V_G_ of Zone‐II transient, respectively. The amount of photocurrent is fixed at ±0.75 nA and the distribution and direction (positive red, negative blue) of photocurrent generated at 0.5 and −0.5 V_D_ are shown. d–f) Photocurrent measured at −6, −1, and −0.9 V_G_ under −1 V_D_ of Zone‐I p–n junction, respectively. The amount of photocurrent is fixed at −1.8 nA (blue). g–i) Photocurrent measured at −0.5, −0.3, and −0.2 V_G_ under 1 V_D_ of Zone‐III enhanced n–n junction before annealing (Figure S2, Supporting Information), respectively. The amount of photocurrent is fixed at 1.5 nA (red). The certain gate bias was determined to observe the change in the position of the photocurrent at the start of tunneling at the junction.

**Figure** **3**a shows that a strong photocurrent (≈1 nA) is only observed across the contact line of WS_2_ and WSe_2_, not in the overlapping junction area. The direction of photocurrent generation tends to extend from the contact line to the center of the WS_2_ sheet. Because the MoS_2_ layer is grounded and the drain bias is only applied on the WSe_2_ layer, the contact line with a low potential barrier in the reverse bias direction shows active photocurrent generation. As shown in Figure 3c, positive 0.5 V_D_ generates the photocurrent only at the WS_2_ and MoS_2_ contact line. These results imply that the external *V*_D_ changes the thickness and the position of the depletion layer. The thickness of the depletion layer can be verified by $w\approx \sqrt{V_{\mathrm{bi}}\pm V_{D}}$ as a function of the external *V*_D_.^[^ ^22^, ^34^ ^]^ Therefore, as shown in Figure 3b, two different heterojunction contact lines at 0 V_D_ generated photocurrents in different directions and gradually disappeared from the center of WS_2_ layer. The depletion layer thickness and the depletion layer ratio in the heterojunction structure can be determined from $w_{\mathrm{Total}}\approx \sqrt{\left(\frac{1}{N_{A}^{\prime }}+\frac{1}{N_{D}^{\prime }}\right)}$ and $\frac{w_{n}}{w_{p}}=\frac{N_{A}^{\prime }}{N_{D}^{\prime }}\;$ considering the equilibrium state of the junction, respectively.^[^ ^34^ ^]^ The *N*′_A_ of WSe_2_ (*N*′_D_ of MoS_2_) is fixed and the *N*′_D_ of WS_2_ can be controlled by electrostatic doping as detailed in Section S7 (Supporting Information).^[^ ^22^ ^]^ As the value of *N*′_D_ increases, the thickness *w*_n_ of the depletion layer becomes insufficient due to the decrease of *w*_Total_, so that the photocurrent generated by the drain bias in the WS_2_ layer increases more than the photocurrent generated by the built‐in barrier. This phenomenon is consistent with the photocurrent distribution formed in a single 2D material‐based phototransistor.^[^ ^36^ ^]^ We also performed the photocurrent mapping as a function of gate voltage under the two different diode directions as p–n junction and n–n junction. As shown in Figure 3d,g, we found the optimized diode biasing conditions as follows: the first diode condition requires −6 V_G_ and −1 V_D_ and the second diode condition requires −0.5 V_G_ and 1 V_D_. In Figure 3d, each heterojunction area generates the photocurrent (1.9–1.5 nA, blue) originated by photovoltaic effect (PVe), and the photocurrent (under 1 nA, green) induced by photoconductive effect (PCe) were generated on the contact line across the junction by the drain bias applied on the WS_2_ layer.^[^ ^37^ ^]^ On the other hand, the n–n junction in Figure 3g shows a lower unit photocurrent than that of the p–n junction due to the weak depletion effect. These results suggest that the low unit photocurrent is effective for electron extraction but hole extraction is not sufficient compared to p–n junction. Under −1 and −0.9 V_G_ conditions in Figure 3e,f, the photocurrent induced by PVe decreased due to the decrease in depletion width, while the photocurrent induced by PCe increased by the tunneling effect (≈10^9^ Ω) rather than by the rectification behavior (≈10^12^ Ω). When the Fermi level of WS_2_ is close enough to the conduction band, the MoS_2_ contact region forms the second diode barrier. As shown in Figure 3h,i, the Fermi level of a fully degenerated WS_2_ in the conduction band causes photocurrent diffusion from the contact line as detailed in Section S8 (Supporting Information). Furthermore, the initial pinch‐off effect (<0.5 V_D_) at the p–n^++^–n^+^ transistor of WSe_2_–WS_2_–MoS_2_ (relative quasi p–n–p) allows us to observe the sufficient n‐type degeneration of WS_2_ as described in Section S9 (Supporting Information).

Thus, we have demonstrated that the main photocurrent in the 2D heterojunction can be controlled by the applied gate voltage. If the *V*_bi_ is large enough to overcome the exciton coupling energy, sufficient photocurrent generation is possible without an external bias. However, when the *V*_bi_ is low, photocurrent generation needs to be assisted by an external applied bias.

D* is a very important figure of merit for photodetectors. To derive D*, the noise equivalent power (NEP) of the photodetector should be considered. In the case of phototransistors, however, the accurate measurement of noise current is very difficult due to the measurement limits. The noise current in photodetectors can be estimated from the sum of Johnson noise (thermal), shot noise (bias) and flicker noise (bias and frequency) as detailed in Section S10 (Supporting Information).^[^ ^4^, ^38^, ^39^ ^]^ We can convert the dark current as a function of gate bias into shot noise and flicker noise. Generally, shot noise tend to increase with increasing dark current. Shot noise current *i*_SN_ can be extracted from the dark current using the following equation, $i_{\mathrm{SN}}=\sqrt{2\cdot q\cdot I_{\mathrm{dark}}}\;$where *I*_dark_ is the dark current of the device.^[^ ^40^, ^41^, ^42^ ^]^ We can also extract the flicker noise current through fitting following the relationship (*1*/*f*)*^*α*^* in noise power density (A^2^ Hz^−1^).^[^ ^8^, ^43^, ^44^ ^]^ However, to date, there is no optimal method for measuring the exact flicker noise current in a 2D‐based phototransistor. Generally, the reported 2D‐based phototransistors have been used the shot noise current estimated from the dark current to calculate the D* using the equation, D* = (*R* · *A* ^1/2^) /(2 · *q* · *I*_dark_)^1/2^, where *A* is the active area of the device and *q* is the electron charge.^[^ ^41^, ^42^, ^45^ ^]^ However, when using the shot noise current to estimate D*, it is possible that it can be overestimated by several orders of magnitude compared to flicker noise current. To eliminate this problem, we verified the origin of phototransistor flicker noise current as a function of gate voltage.

**Figure 4:**
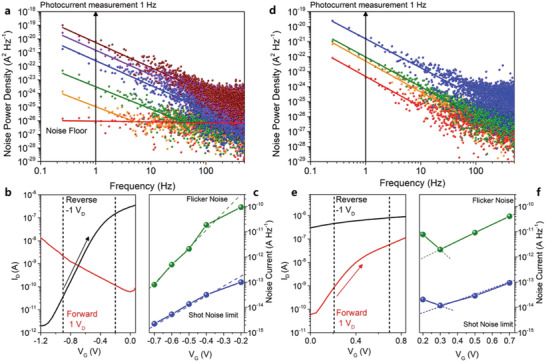
Flicker noise measurement and shot noise estimation as a function of charge balance from the gate bias at first and second diode conditions. a) Noise power density as a function of Hertz from −0.7 V_G_ (red) to −0.2 V_G_ (brown) at −1 V_D_. d) Noise power density as a function of Hertz from 0.2 V_G_ (orange) and 0.3 V_G_ (red) to 0.7 (blue) at 1 V_D_. a,d) Linear fitted solid line for a guide to the eye under 100 Hz. Photocurrent measurement at 1 Hz  chopped condition was processed and black arrow indicate noise power density level. b,e) Transfer curve at ±1 V_D_ in transient gate bias range of diode 1 and diode 2, respectively. c,f) Flicker (green dot line) and shot noise (blue dot line) at −1 and 1 V_D_ reverse bias of p–n and n–n junctions.

**Figure** **4**a,d shows the variation of the flicker noise current in the p–n WSe_2_–WS_2_ junction diode and the n–n WS_2_–MoS_2_ junction diode, respectively. Figure 4b shows the transfer curve of the p–n junction diode measured as a function of gate bias at forward (+1 V_G_) and reverse (−1 V_G_) bias under dark condition. The dark current as a function of gate bias can be converted into the shot noise and flicker noise currents. In Figure 4a, the current level of 10^−10^ A at −0.7 V_G_ has a noise floor behavior around 10^−26^ A^2^ Hz^−1^.

A current level of 10^−9^ A at −0.6 V_G_ begins to show the flicker noise current from the sub‐log‐linear fit (*1*/*f*)*^*α*^*.^[^ ^8^, ^21^, ^43^, ^46^ ^]^ Figure 4c shows a comparison of the flicker noise and the shot noise current as a function of gate bias. We extracted the flicker noise current at 1 Hz. The flicker noise magnitude is ten times higher than the shot noise in the low dark current range of 10^−10^ A. As the dark current increases, the flicker noise current gradually increases to 1000 times more than the shot noise. On the other hands, the dark current (6 × 10^−11^ A) of the n–n junction diode is much higher than the dark current (2 × 10^−12^ A) of the p–n junction diode as shown in Figure 4e because the rectification ratio of n–n junction diode is poor as described in Section S3 (Supporting Information). The behavior of flicker noise current in the n–n junction device is similar to that of the p–n junction diode.

Interestingly, the flicker noise current measured at 0.2 V_G_, as shown in Figure 4f, appears to be inversely proportional to the dark current. This can be attributed from the hysteresis by the charge balance transient observed in the transfer curve as detailed in Section S11 (Supporting Information). The vacancies and chemical traps on the surface of the channel can affect the magnitude of flicker noise current, and it can be expected that the gate field effect diode will depend heavily on the junction capacitance as a function of the depletion layer thickness as detailed in Section S13 (Supporting Information).

We also measured the time‐resolved photocurrent response (TRPR) at each optimized diode condition (diode 1: −3 V_G_; diode 2: 0 V_G_) with a 1 Hz  chopped 405 nm  laser (beam size: 0.5 µm) as a function of incident light power to obtain the responsivity (detailed in Section S12 in the Supporting Information). The responsivity and TRPR measured under reverse bias conditions of the first diode were 3.82 × 10^6^A W^−1^and 9 ms. Additionally, the D* can be calculated using the following formula, $D^{*}=\;R\sqrt{A\cdot B}/\;i_{\mathrm{FN}}=\sqrt{A\cdot B}\;/\mathrm{NEP}$, where *R* is the responsivity, *A* is the active area of detector, *B* is the bandwidth, *i*_FN_ is the flicker noise current, and NEP is *i*_FN_/*R*.^[^ ^45^ ^]^ The calculated D* is 5.98 × 10^15^ cm Hz^1/2^ W^−1^, which is much higher than the value reported for previous 2D heterojunction devices.^[^ ^5^, ^8^, ^46^, ^47^ ^]^

## 3 Conclusion

In summary, we have demonstrated a multifunctional 2D heterojunction p–n–n phototransistor where the charge balance can be controlled via electrostatic doping to pinpoint the origin of photocurrent and noise current in various heterojunction devices. Through photocurrent mapping, we identified two kinds of photocurrent generation mechanisms. First, the drain bias applied to the WS_2_ channel generates photocurrent induced by PCe on the TMDC junction line. Second, the built‐in potential formed in the p–n heterojunction generates the photocurrent induced by PVe at TMDC junction area. We also analyzed the relationship between the dark current and the flicker and shot noise currents of the phototransistor. The calculated responsivity and D* obtained from the optimized charge balance to form the ideal diode was 3.82 × 10^6^ A W^−1^ and 5.98 × 10^15^ cm Hz^1/2^ W^−1^ under 1 Hz  chopped light, respectively. A TRPR of 9 ms  in low optical energy (20 nW cm^−2^) was also achieved. The results demonstrate that the charge balance in van der Waals heterojunction is very important for optoelectronic applications. This study reveals that even if the charge densities of the active materials of the layered structures are not perfectly matched, it is still possible to create an optoelectronic device having excellent characteristics by tuning the charge balance through the gate voltage.

## Conflict of Interest

The authors declare no conflict of interest.

## Supporting information

Supporting Information
