# Supplementary material for: Probing the Importance of Charge Balance and Noise Current in WSe2/WS2/MoS2 van der Waals Heterojunction Phototransistors by Selective Electrostatic Doping
Source: Adv Sci (Weinh). 2020 Aug 18;7(19):2001475. doi: 10.1002/advs.202001475 (PMC7539183; doi:10.1002/advs.202001475)
Supplement: Supplementary file 1 — Supporting Information [file ADVS-7-2001475-s001.pdf]

© 2020 Wiley-VCH GmbH

ADVANCED SCIENCE
Open Access

Supporting Information

for *Adv. Sci.*, DOI: 10.1002/advs.202001475

Probing the Importance of Charge Balance and Noise Current in $WSe_2/WS_2/MoS_2$ van der Waals Heterojunction Phototransistors by Selective Electrostatic Doping

*Hyun-Soo Ra, Min-Hye Jeong, Taegeun Yoon, Seungsoo Kim, Young Jae Song, and Jong-Soo Lee**

Supporting Information

Title: Probing the importance of Charge Balance and Noise Current in $WSe_2/WS_2/MoS_2$ van der Waals Heterojunction Phototransistors by selective electrostatic doping

Hyun-Soo Ra, Min-Hye Jeong, Taegeun Yoon, Seungsoo Kim, Young Jae Song and Jong-Soo Lee *

Dr. Hyun-Soo Ra, Min-Hye Jeong, Prof. Jong-Soo Lee
Department of Energy Science & Engineering
Daegu Gyeongbuk Institute of Science and Technology (DGIST)
Daegu 42988, Republic of Korea
E-mail: jslee@dgist.ac.kr
Seungsoo Kim
Department of Nano Engineering, Sungkyunkwan University (SKKU), Suwon, Korea
Taegeun Yoon, Prof. Young Jae Song
SKKU Advanced Institute of Nano Technology (SAINT), Sungkyunkwan University (SKKU), Suwon, Korea
Department of Nano Engineering, Sungkyunkwan University (SKKU), Suwon, Korea

Keywords: transition metal dichalcogenides, heterojunction, electrostatic doping, scanning photocurrent mapping, noise current

Contents

Methods
Supporting section 1: 2D thickness measurement using AFM
Supporting section 2: WSe_2 annealing to enhance p-type property
Supporting section 3: 2 different diode characteristics
Supporting section 4: The p-n junction photovoltaic effect analysis
Supporting section 5: Laser beam size for scanning photocurrent mapping
Supporting section 6: Transient zone output curve.
Supporting section 7: Depletion width as a function of charge balance
Supporting section 8: Tunneling leakage current effect
Supporting section 9: Transistor application like quasi p-n-p.
Supporting section 10: Flicker Noise
Supporting section 11: Electrical hysteresis of diode transient gate region
Supporting section 12: Time-Resolved Photocurrent Response at p-n and n-n junction
Supporting section 13: The perspective of junction capacitance

Methods

Device fabrication. We prepared polyvinyl alcohol (PVA) as water-soluble layer and poly (methyl methacrylate) (PMMA, 950K) thin film as acetone soluble layer on SiO_2 wafer for 2D TMDCs (HQ graphene) transfer. Homemade optical microscopy equipped with ultra-long working distance lens (ULWD, Motic) and heating plate (130 °C) was used to align 2D materials. The fine electrodes on 2D materials were fabricated using an acceleration voltage of 20 keV from Raith 150 TWO EBL system. Free patterned gate- and source-drain electrodes were fabricated as the thickness of Titanium (5 nm)-Gold (20 nm), Titanium (10 nm)-Gold (40 nm) using electron beam evaporation system (SORONA), respectively. 2D material active area was proceeded using reactive ion etching (RIE) system as O_2 and CHF_3 gases with RF 80W condition.

Electrical and scanning photocurrent mapping measurement. Homemade LabVIEW program was used to control three probe transistor measurement set-up using Keithley 2636B (GPIB cable) and scanning photocurrent mapping system (RS232 cable). Laser (405 nm, BLM405TA-80R) scanning system is operated by a single protected silver mirror (450~1000 nm) with a motorized x-y scan controller. Laser scanner resolution is < 20 nm. 100X objective ULWD lens was used for *in-situ* current probing. All measurements were performed in the darkroom. All mapping data was precisely analyzed using the origin plot program with interval 150 step color. Time-reolved photocurrent response (TRPR) was collected using precise time-interval measurement system (buffer time measurement) and laser transistor-transistor logic (TTL) modulation through function generator Hz controller.

Noise current measurement. LabVIEW program (buffer time measurement) to operate the Keithley 2636B was designed for 1000 Hz sampling and 5 second current tracing. For the accurate current measurement, the current level (nA, μA) in LabVIEW was fixed to minimize noise. All measurements were conducted with RF current measurement cable and ground handing in measurement condition. We calculated detectivity through flicker noise fitting $(1/f)^{\alpha}$. All noise power density ($A^2\ Hz^{-1}$) was extracted for each Hz using fast Fourier transform (FFT) from raw noise current tracing data. The noise current ($A\ Hz^{-1/2}$) can be

obtained by taking root in the noise power density. The responsivity of 1 Hz modulation was considered to calculate detectivity using the noise current (A).[1, 2]

Kelvin probe force microscope (KPFM) measurement. We used model NX 10 from the Park system company. KPFM measurement was conducted in non-contact mode/EFM mode. We used the NSC36/Cr-Au tip as an AFM probe. Keithley 2410 was used for external gate voltage source and ground unification with NX 10, and work function was measured through a KPFM tip. HOPG was used to calibrate the work function between the KPFM tip and the material. We acquired images through line scan method for precise work function measurement, and conducted more than 20 samplings.

Supporting section 1: 2D thickness measurement using AFM

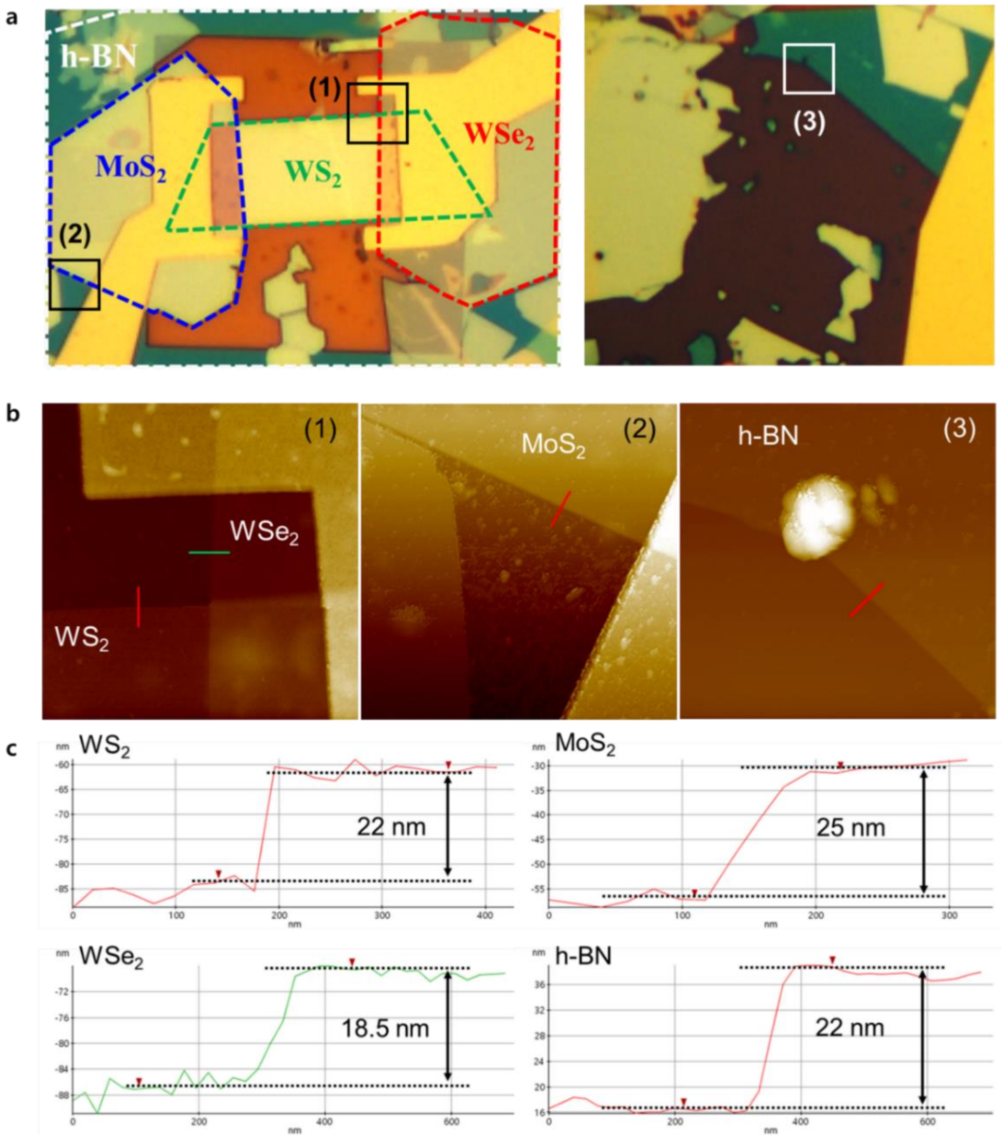

Figure S1. a) is optical microscope image of our multifunctional 2D heterojunction phototransistor for checking AFM area (1-3 section). b) is AFM mapping image at each section with WS_2, MoS_2, WSe_2, and h-BN. c) is AFM line profile at each 2D materials.

We chose bulk TMDCs of around 20 nm thickness to enhance absorption property. In additionally, we referred the *C.H. Lee et al* report external quantum efficiency of 2 % (monolayer junction) and 50 % (multilayer > 9 nm junction) in MoS_2-WSe_2.[3] h-BN of 22 nm thickness was used for efficiently current (μA) blocking as the gate dielectric layer.[3, 4]

Supporting section 2: WSe_2 annealing to enhance p-type property

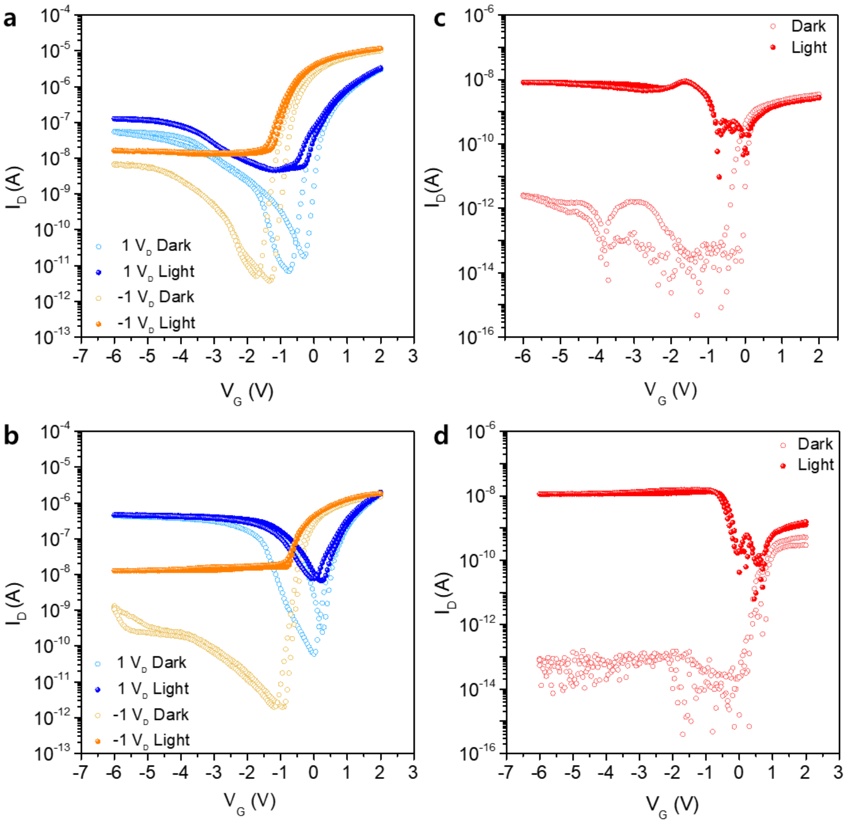

Figure S2.1. a) and b) is transfer curve with white incident light of 52 μW cm^{-1} as a function of gate bias double sweep and at forward-reverse drain bias of ±1 V_D. c) and d) is transfer curve to understand photovoltaic effect under the same measurement condition at 0 V_D bias. a) and c) is just fabricated device. After annealing 200 °C in atmosphere, b) and d) show p-type doped and optimum charge balance tendency.

Hole enhancement of WSe_2 through annealing process

We annealed to enhance the hole properties of WSe_2. Annealing 200 °C in atmosphere can control the surface acceptor by forming a self-limiting oxide layer (WO_x) of WSe_2 on the surface. We found that the annealing time of about 1 hours reached the limit of the oxide layer and the characteristics were not changed anymore. Furthermore, the stronger hole properties can be obtained in the ozone O_3 and 100 °C temperature environment, but the annealing conditions described above were used to evaluate the device characteristics in the air atmosphere.[5, 6] We observed that the hole characteristics

of WSe_2 sufficiently strengthened after annealing improved the rectification characteristics (1 order magnitude current from 10^{-8} to 10^{-9} A) by forming a sufficient depletion layer thickness at the interface with WS_2. The photovoltaic effect also lowers the rectified current characteristics, and we can see that a more efficient p-n junction is formed due to the increased amount of photocurrent and lower rectified current level from 10^{-12} to 10^{-13} A. On the other hand, the second diode rectification characteristic formed at the n-n junction tends to decrease (1 order magnitude current from 10^{-11} to 10^{-10} A).

Tunable carrier (electron) concentration of WS_2 as a function of a gate bias.

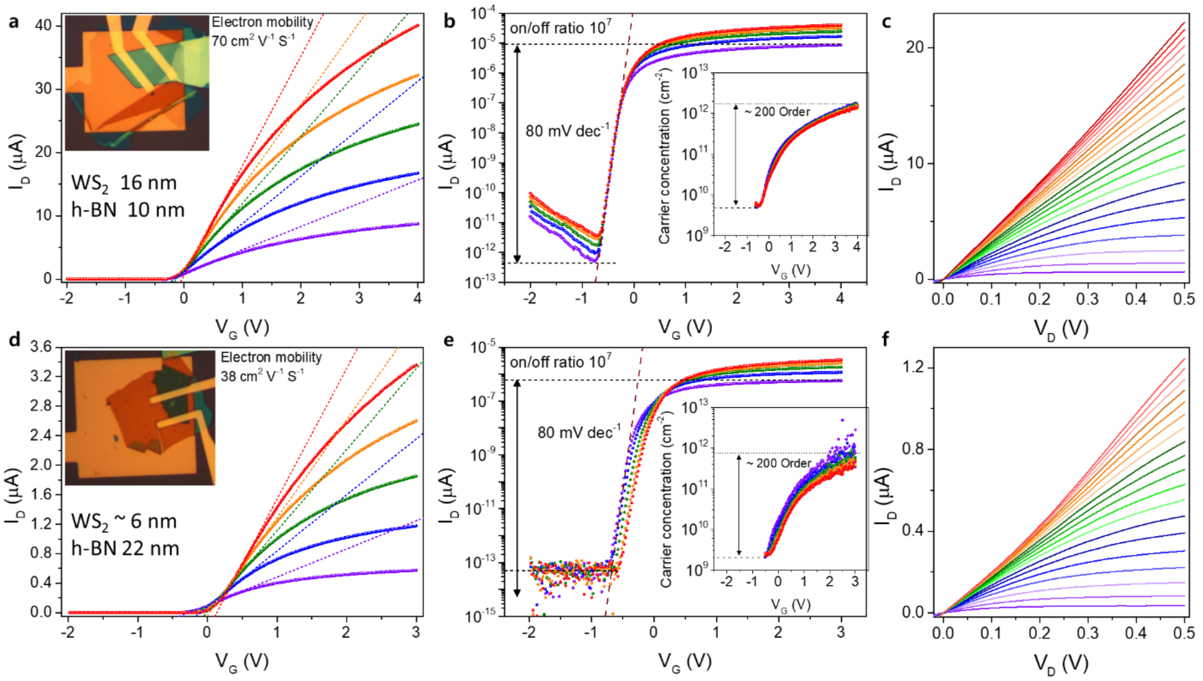

Figure S2.2. a) and d) is linear transfer curve of 16 nm, 6 nm WS_2 as a function of V_D from 0.1 (violet) to 0.5 V (red) with 0.1 step, respectively. b) and e) logarithm transfer curve for on/off ration and subthreshold voltage. Inset show tunable 200 order magnitude of carrier concentration (cm^{-1}). c) and f) show output curve as a function of V_G from -1 (violet) to 3 V (red), which is operation range (high gradient region, g_m) in transfer curve.

We measured the WS_2 FETs of 16 and 6 nm, respectively, in order to investigate the basic transport properties and the change of carrier concentration as a function of a gate bias. First, the mobility of WS_2 can be calculated by the following equation:

$$\mu = \left(\frac{L \cdot g_m}{W \cdot C_{h-BN} \cdot V_{DS}}\right)$$

Where L and W is length and width of channel, g_m is gradient (I_D/V_G), C_{h-BN} is capacitance of h-BN layer, V_{DS} is drain-source voltage bias. WS_2 of 16 nm has 70 $cm^2\ V^{-1}\ S^{-1}$ and WS_2 of 6 nm has 38 $cm^2\ V^{-1}\ S^{-1}$. The on/off ratio of WS_2 was measured to be ~10^7, and S.S. was 80 mV dec^{-1}, which was confirmed to be a very good device considering the limit of 60 mV dec^{-1} at room temperature. Based on the mobility of the device, we calculated the carrier concentration through the following equation:

$$n = \left(\frac{\sigma}{e \cdot \mu}\right) = \left(\frac{L \cdot I_{DS}}{W \cdot e \cdot \mu \cdot V_{DS}}\right) = \left(\frac{I_{DS} \cdot C_{h-BN}}{e \cdot g_m}\right) = \frac{C_{h-BN}(V_G - V_{Th})}{e}$$

Where σ is conductivity, e is electron (coulomb), and I_{DS} is drain-source current.

The WS_2 channel carrier concentration of 16 nm was measured to be from 4.8×10^{10} cm^{-2} at -0.5 V_G to 1.6×10^{13} cm^2 at 4 V_G. The WS_2 channel carrier concentration of 6 nm was measured to be fro m 2×10^{10} cm^{-2} at -0.5 V_G to 6×10^{13} cm^{-2} at 3 V_G. Two WS_2 channels of different thickness have a suffi ciently wide bandgap, so the carrier concentration is determined by the capacitance of the h-BN dielect ric. The evidence is that the same carrier concentration ratio is observed as like 200 order magnitude.[3, 7, 8]

Kelvin probe force microscope (KPFM) measurement to understand the work function modulation as a function of gate bias

We measured KPFM to demonstrate the electrical field effect applied only to WS_2. As shown in Figure S2.3a, we line scan the central area of the $MoS_2/WS_2/WSe_2$ devices to increase the reliability of the KPFM and observe modulation in work function. We matched the common ground in the measurement equipment Keithley to NX10 (KPFM) ground, and the field effect was applied to the sample by connecting the Keithley voltage terminal to the gate pad of the sample. We observed the work function modulation through the reading probe (after HOPG reference) on the surface of the

sample, which is receiving the overall field effect. Figure S2.3c shows the result of observing the modulation in the work function at each gate bias by performing line scan more than 20 times. We can clearly observe the mapping result that the work function modulates according to each gate bias. Figure S2.3(d) shows the work function in each material (MoS_2, WS_2, and WSe_2) depending on the applied gate voltage from -4V_G to 2V_G.

This modulation was the result of the Fermi level being theoretically matched with the direction of movement by the electrical field effect, and the work function modulation history was clearly observed. We could not find the gate leakage current during KPFM measurement. (stable 10^{-10} A level)

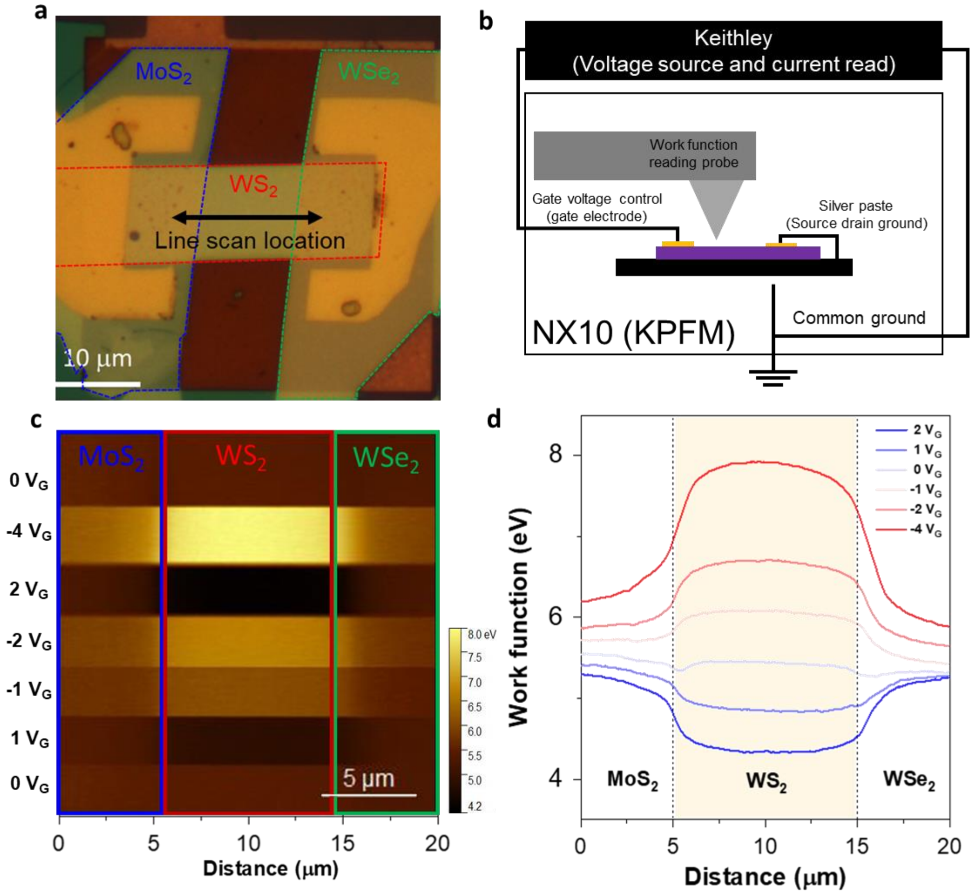

Figure S2.3 (a) MoS_2/WS_2/WSe_2 device optical image and KPFM line scan location, (b) schematic diagram and electrical circuit drawing of Keithley and NX10 (KPFM), (c) KPFM line scan data mapping as a function of gate bias, (d) Work function of MoS_2, WS_2 and WSe_2 depending on the applied gate bias.

The energy results obtained from the line scan accumulated more than 20 times are averaged and shown in the following table S1 and S2.

	-4 V_G	-2 V_G	-1 V_G	0 V_G (Equilibrium)	1 V_G	2 V_G
WS_2	7.91 eV	6.71 eV	6.08 eV	5.42 eV	4.85 eV	4.27 eV
MoS_2	6.36 eV	5.92 eV	5.72 eV	5.50 eV	5.34 eV	5.16 eV
WSe_2	6.00 eV	5.71 eV	5.47 eV	5.28 eV	5.25 eV	5.19 eV

Table S1. TMDCs work function modulation history by the electrical field effect.

The difference between the work function of WS_2 and MoS_2 is 80 meV, and the work function of WS_2 is lower than that of MoS_2, and it is observed to be similar to 94.82 meV of the literature value. [9] The difference between the work function of WS_2 and WSe_2 was measured to be 220 meV, and the work function of WSe_2 is lower than that of WS_2, and this trend is similar to the literature value of 113 meV. [10] As in the cascade band alignment shown in our main Figure 2c, the work function of MoS_2, WS_2, and WSe_2 were measured in order of magnitude. It can be seen that the difference between our measurement work function and the literature value comes from the thickness of TMDCs, and the difference can be more than 80 meV. [11]

	Negative ΔV_G (-4 V ~ 0 V)			Positive ΔV_G (0 V ~ 2 V)		AVG.
	ΔV_G = -2	ΔV_G = -1	ΔV_G = -1	ΔV_G = 1	ΔV_G = 1	-
WS_2	1.20 eV	0.63 eV	0.66 eV	-0.57 eV	-0.58 eV	0.60 eV
MoS_2	0.44 eV	0.2 eV	0.22 eV	-0.16 eV	-0.18 eV	0.20 eV
WSe_2	0.29 eV	0.24 eV	0.19 eV	-0.03 eV	-0.06 eV	0.13 eV

Table S2. Work function modulation according to the positive and negative unit gate bias (1 V) for each material.

We added the table S2 to quantify how the work function of each material modulates with the unit gate bias (1 V). In the case of WS_2, the negative ΔV_G and the positive ΔV_G showed 0.66 eV and -0.57 eV (avg. 0.60 eV). It has similar work function modulations with linear behavior. However, in the case of

MoS_2, the negative ΔV_G and the positive ΔV_G showed 0.22 eV and -0.16 eV (avg. 0.20 eV) with different work function modulation. Similarly, for WSe_2, the work function modulation at negative ΔV_G and the positive ΔV was measured significantly different from each other, such as ~0.19 eV and -0.03 eV (avg. 0.13 eV) at unit gate bias (1V). The magnitude (%) of the work function modulation in MoS_2 and WSe_2 differed by up to 33.3% and 21.6%, respectively, compared to WS_2 (100%).

It is calculated from the following equation;

(avg. work function of MoS_2 or WSe_2 or WS_2) / (avg. work function of WS_2) × 100

Therefore, Considering WS_2's work function modulation size and symmetry in positive ΔV_G and negative ΔV_G, we can understand that the field effect is mainly applied to WS_2.

It can be understood that the large asymmetry in the negative ΔV_G and the positive ΔV_G is caused by the charge transfer caused by the difference in the Fermi energy alignment of MoS_2-WS_2 and WSe_2-WS_2. For this reason, it is possible to support the generation of asymmetric ΔV_G.

Supporting section 3: 2 different diode characteristics

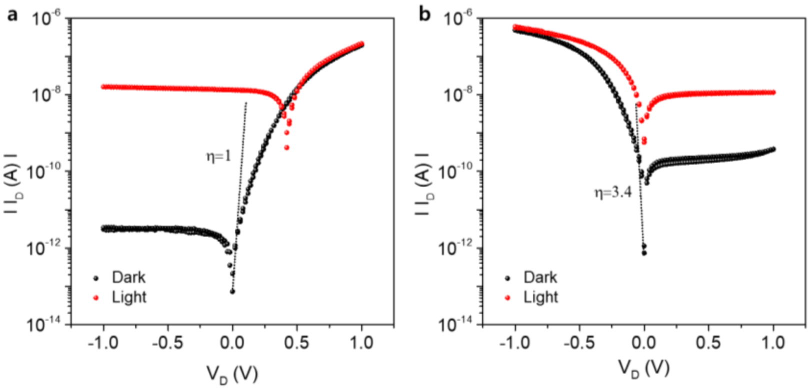

Figure S3. a) is output curve as a function of drain bias at -1.3 V_G of p-n junction region. This output curve has ideal factor ~1 and rectification ratio of 5.7 x 10^4 within -1 V_D to 1 V_D. b) is output curve as a function of drain bias at 0.3 V_G of n-n junction region. This output curve has ideal factor ~3.4 and rectification ratio of 1.3 x 10^3 within -1 V_D to 1 V_D.

The ideal factor (η) was extracted from the graph using the following equation:

$$\eta = \left(\frac{q}{k_B T}\right)\left(\frac{dV_D}{d\ln I_D}\right)$$

Supporting section 4: The p-n junction photovoltaic effect analysis

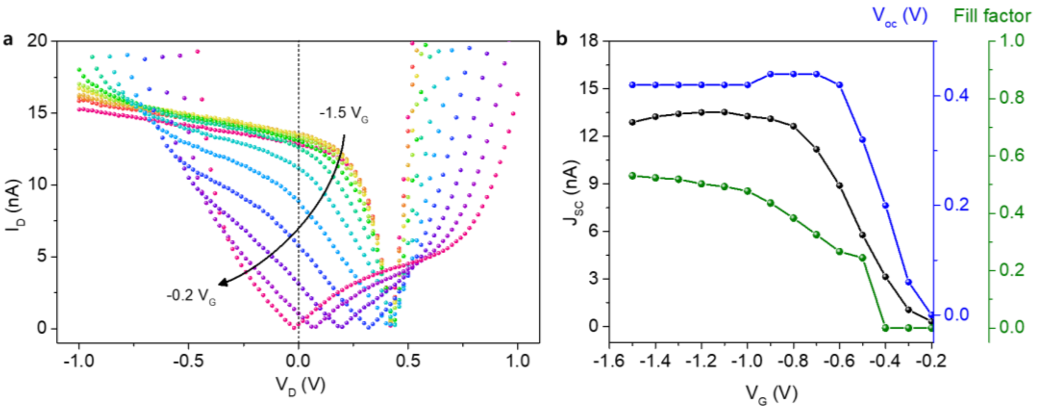

Figure S4. a) is output curve as a function of drain bias with gate bias from -1.5 V_G to -0.2 V_G under white incident light of 52 μW cm^{-1} (not one sun). b) is photovoltaic effect characteristics as a following factor; short-circuit current (J_{SC}), open-circuit voltage (V_{OC}), and fill factor. Highest efficiency is J_{SC} = 13.52 nA, V_{OC} = 0.44 V, and FF = 0.53.

Supporting section 5: Laser beam size for scanning photocurrent mapping

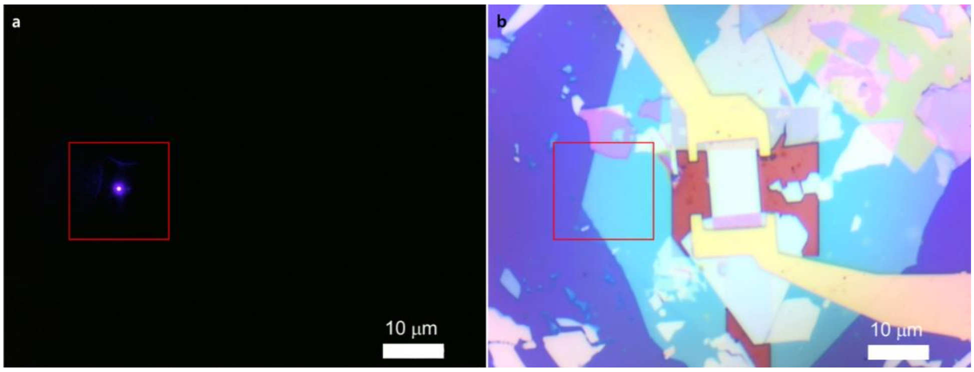

Figure S5. a) is 405 nm laser beam size image taken by charge coupled device (CCD) camera equipped by photocurrent mapping setup. b) is optical microscope image for matching with (a) image. Evidence of measuring beam size at the same point through a reflective contour.

Beam spot diameter of objective lens, Spot Diameter = 1.22 x λ / N.A

We used APO objective lens (ULWD stardard 100x) for focusing 405 nm laser beam.

Lens information : Magnification 100x, Numerical aperture (N.A.) = 0.550,

Working distance (W. D.) = 13.0 (mm), Resolution = 0.5 (um)

Then, a diffraction limit of 898 nm diameter (~ 0.9 μm) of 405 nm laser beam size, active area of responsivity is 2.5 μm^2

Supporting section 6: Transient zone output curve.

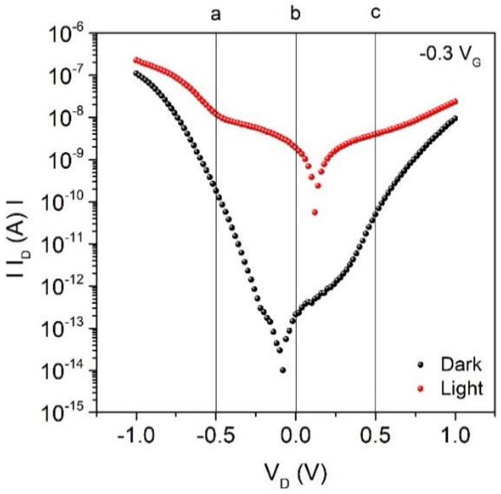

Figure S6. Transient output curve as a function of -0.3 V_G. Indexed a-c at -0.5, 0, 0.5 V_D correspond with main Figures 3 a-c) of photocurrent mapping data

Supporting section 7: Depletion width as a function of charge balance

We approach heterojunction depletion width analysis of TMDCs as similar doping concepts of commercialized silicon. TMDCs are MX_2 structures (Hexagonal) composed of various transition metals (Mo, W, Sn, Re, Nb, Zr, Hf…) and chalcogenide (S, Se, Te…) compounds. It is similar to the silicon crystal structure in which various types of atoms (Group Ⅲ and Ⅴ atom) are doped to change their properties. As the syntheses and characterization of materials (p-WSe_2, n-MoS_2, p-$MoTe_2$, and n-WS_2) having various charge characteristics of such 2D semiconductors are being actively carried out, the most important challenge we have now is to control the properties of microdoping for finely tuning charge balance.

In this study, we demonstrate the characteristics of the photocurrent and photoelectric properties of the two-dimensional heterojunction properties of charge balance by controlling the Fermi level of very thin 2D materials using the gate field effect. The thickness (w) and the ratio (w_n/w_p) of the depletion layer generated in the p-n junction structure applied in the silicon semiconductor will be briefly described using the following equation.[12]

$$w_{Total} = w_n + w_p = \sqrt{\left[\frac{2\varepsilon V_j(n_p + n_e)}{q n_p n_e}\right]}$$

Where n_p is the hole concentration of WSe_2, n_e is the electron concentration of WS_2, ε is the dielectric constant, and V_j is junction voltage in depletion region. Assuming that the n_p value of WSe_2 is unchanged, the thickness of the total depletion layer narrowed by n_e increased due to the gate field effect in WS_2. The thickness of the depletion layer formed on each bonding surface can be derived by the following equation.[12]

$$w_n = \sqrt{\left[\frac{2\varepsilon V_j}{q n_e(1+\frac{n_e}{n_p})}\right]},\ w_p = \sqrt{\left[\frac{2\varepsilon V_j}{q n_p(1+\frac{n_p}{n_e})}\right]}$$

$$\frac{w_n}{w_p} = \frac{n_p}{n_e}$$

When the n_e value of WS_2 is increased, the thickness of the entire depletion layer is decreased and the ratio of WS_2 is also decreased. In addition, the value of the field effect ($\mathbb{E}_{max}$) generated inside the junction by the depleted depletion layer can be expressed by the following equation.[12]

$$\mathbb{E}_{max} = \frac{2V_j}{w_{Total}}$$

When the thickness of the depletion layer is narrowed, the tunneling effect is maximized because the field effect is maximized. The tunneling effect removes the rectification effect from the junction and can explain the phenomenon that the origin position of the photocurrent is shifted to the cause of the single layer WS_2. (Figures 3e, f, h, i)

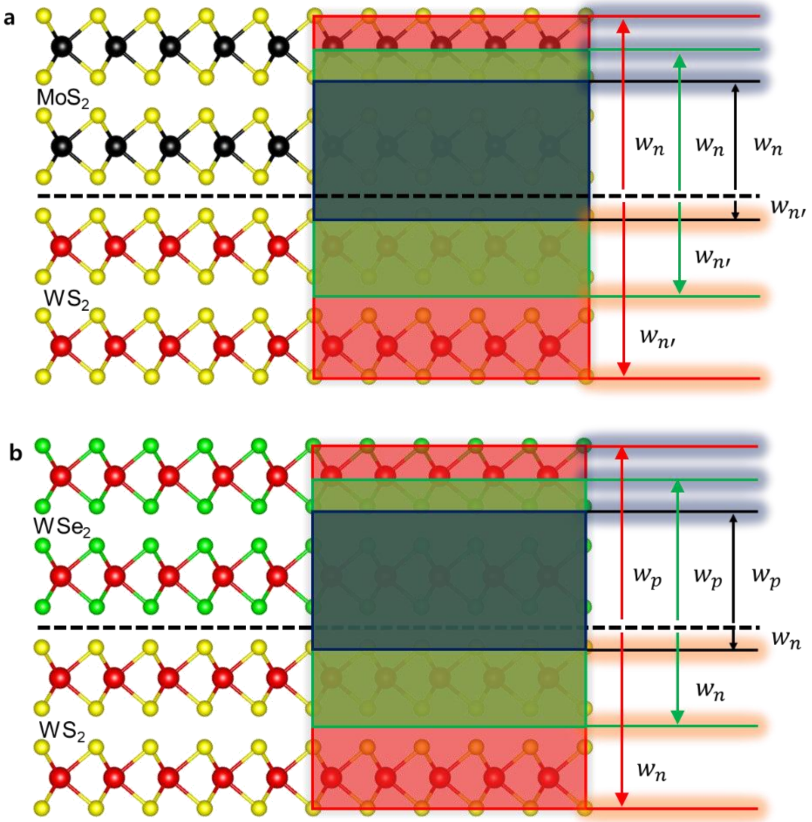

Figure S7. a) is the schematic diagram showing the tendency of the depletion layer ratio formed on each heterojunction (n-n junction of MoS_2-WS_2). The ratio of the electron concentration of MoS_2 (w_n) and WS_2 (relative depletion region, $w_{n'}$) as a function of the gate bias. (b) is the schematic diagram showing the tendency of the depletion layer ratio formed on each heterojunction (p-n junction of WSe_2-WS_2). The ratio of the electron concentration of WSe_2 (w_p) and WS_2 (w_n) as a function of the gate bias.

A depletion layer is formed in the area band represented by the red line when the charge density ratio is the same, and the depletion layer ratio formed in each 2D material is also divided into 1:1. In the gate bias where the charge ratio is broken ($\frac{n_p}{n_e} \neq 1$), the overall thickness reduction of the depletion layer and the ratio of the depletion layer are expressed as shown in Figure S7.

Supporting section 8: Tunneling leakage current effect

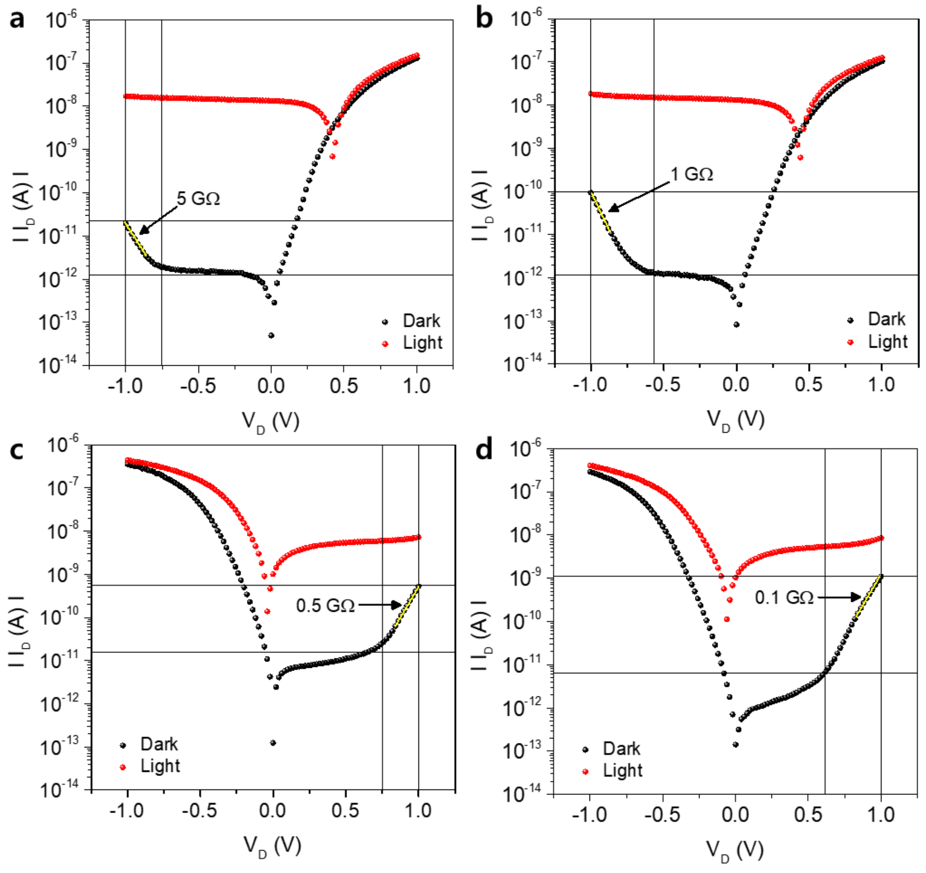

Figure S8. a) and b) are output with leakage effect indicated by yellow line fitting at -1 V_G and -0.8 V_G. c) and d) at 0.1 V_G and -0.1 V_G.

Supporting section 9: Transistor application like quasi p-n-p

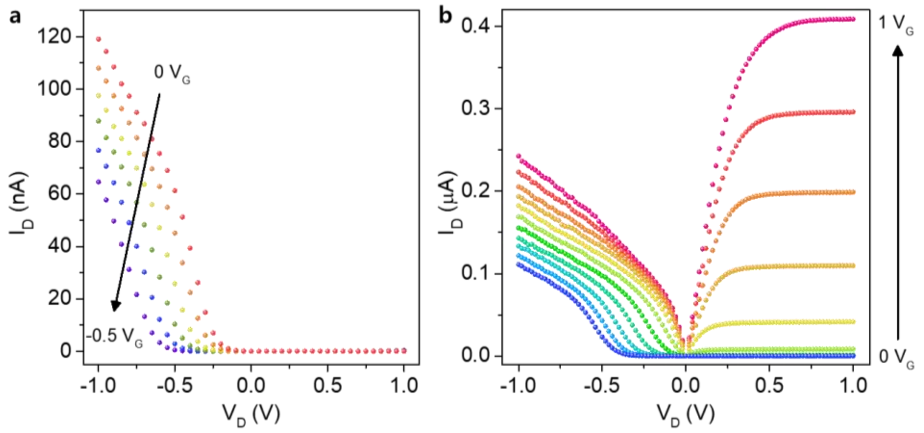

Figure S9. a) second diode characteristics. b) output curve of quasi p-n-p transistor characteristics.

Supporting section 10: Flicker Noise

Noise consist of thermal noise (Johnson noise) as a function of temperature, Shot noise as a function of applied voltage and flicker noise as a function of various variables under 100 Hz, as mentioned in the main manuscript. Johnson noise and Shot noise exist in the form of a floor, regardless of the frequency band. Therefore, Johnson noise can be calculated by the following formula;[13]

$$i_n = \sqrt{\frac{4k_B T \Delta f}{R}}$$

Where k_B is Boltzmann's constant in joules per kelvin, T is absolute temperature, Δf is electrical bandwidth, R is resistance of materials in device. Noise generated when an external voltage is applied to the photodetector has shot noise and flicker noise. Shot noise refers to the current noise that causes a difference in the rate at which an electron enters a material through an electrode. This current noise is created by locally obstructing the current flow due to the contact resistance between the material and the metal electrode and the defects, which occur carrier trap in the material. shot noise can be calculated from the DC signal by the following formula;[13, 14]

$$i_n = \sqrt{2q i_{dark} \Delta f}$$

Where q is electron, i_{dark} is dark current formed by applied DC voltage, and Δf is bandwidth. Using fast Fourier transform (FFT) algorithm obtained by finite sampling of the dark current of photodetector, the method of measuring flicker noise can obtain the noise-frequency spectrum of a sample, which is calculated by the following formula;

$$X_k = \sum_{n=0}^{N-1} x_n e^{\frac{-i2\pi kn}{N}} = \sum_{n=0}^{N-1} x_n (\cos\frac{2\pi kn}{N} + i \sin\frac{2\pi kn}{N})$$

The noise frequency obtained by the FFT can be sampled by setting the rated current level of the Keithley 2636B precision instrument and up to 1000 samples per second (1000 Hz).[15-17] We calculated the Noise Power Density ($A^2\ Hz^{-1}$) of the Figure 4a, b through the following current sampling data of Figure S10.

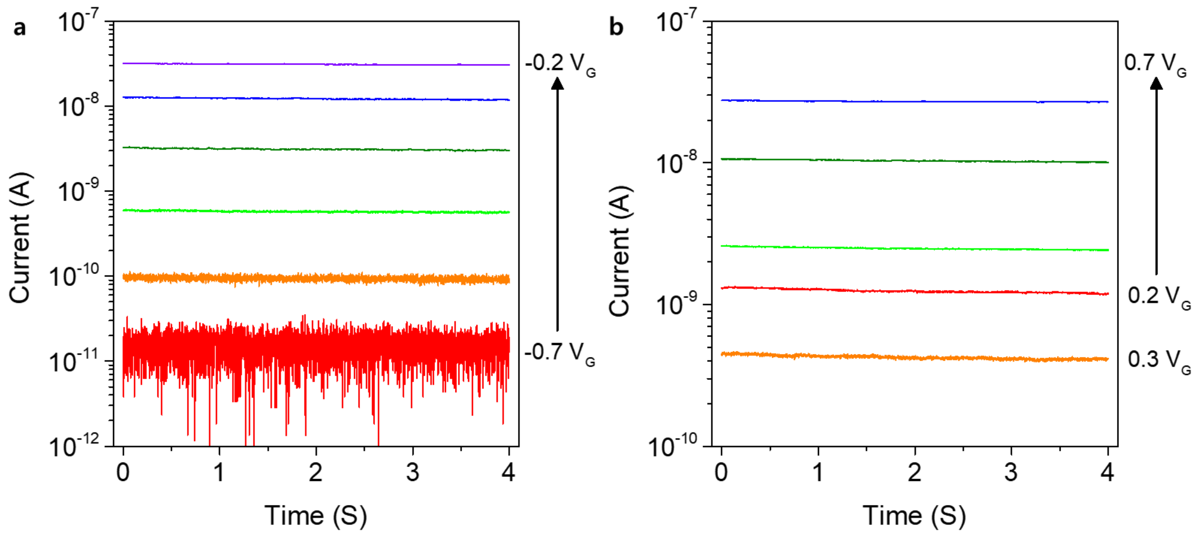

Figure S10. a) is current sampling trace at -1 V_D during 4 second with 0.001 second interval. Gate bias range from -0.7 V_G to -0.2 V_G with 0.1 V_G step. b) is current sampling trace at 1 V_D during 4 second with 0.001 second interval. Gate bias range from 0.2 V_G to 0.7 V_G with 0.1 V_G step.

Supporting section 11: Electrical hysteresis of diode transient gate region

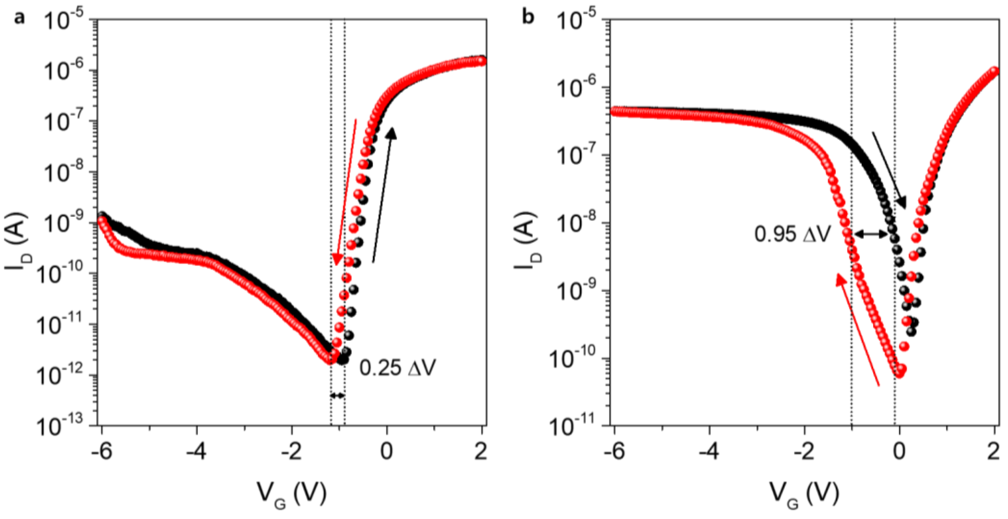

Figure S11. a) is hysteresis analysis with 0.25 ΔV in transfer curve sweep at reverse bias -1 V_D of first diode. b) is hysteresis analysis with 0.95 ΔV in transfer curve sweep at reverse bias 1 V_D of second diode. Red dots are 2 to -6 V_G direction sweep, and black dots is -6 to 2 V_G direction sweep.

Discussion of gate functional hysteresis

As shown in the Figure S11a, the hysteresis caused by the h-BN gate function was hardly observed as 0.25 ΔV. This result is observed because the single crystal h-BN layer and the perfectly flat surface have less mutual interference due to the second van der Waals bond with the other 2D material. However, hysteresis in a specific gate sweep region from -1 V_G to 0 V_G is relatively large as like 0.95 ΔV (380 % increase) in the Figure S11b. This region coincides with the zone 2 transient section shown in Figure 2b. In this section, the value of qV_{bi} is very important in the section where Voc changes abruptly. We can expect that the origin of the hysteresis increasing is caused by the junction capacitor made by the depletion layer thickness change due to the electron concentration change in WS_2. We will discuss the junction capacitance in detail in **Supporting section 13**. Transfer curve measurement condition; sampling with gate voltage sweep of 0.05 V_G interval per 0.04 second.

Supporting section 12: Time-Resolved Photocurrent Response at p-n and n-n junction

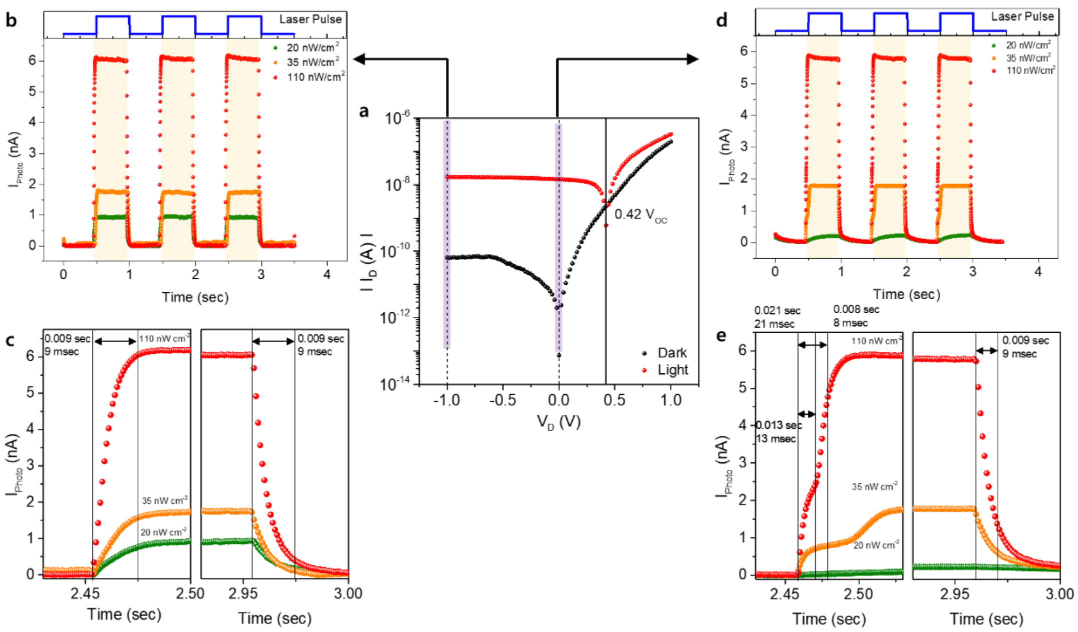

Figure S12.1. a) is output with white incident light of 52 μW cm^{-1} at -3 V_G. Violate region is TRPR measurement region. b) is overall light on-off behavior as a function of different light incident power at -1 V_D. c) is magnified image for time-resolved rising and decay analysis. d) is overall light on-off behavior as a function of different light incident power at 0 V_D. e) is magnified image for time-resolved rising and decay analysis.

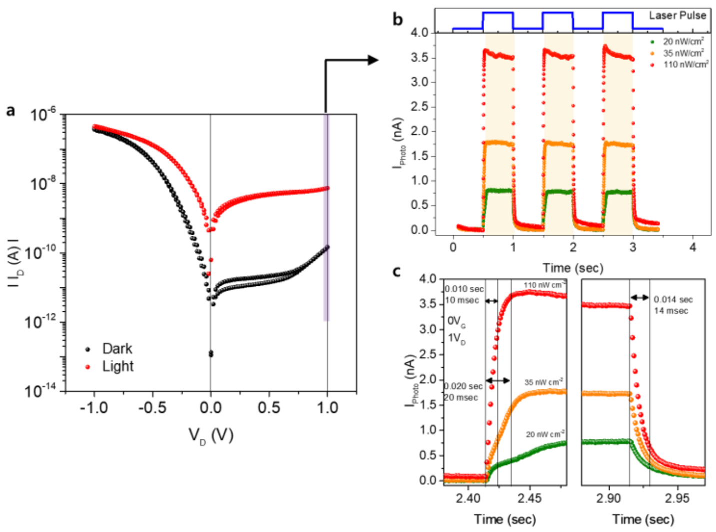

Figure S12.2. a) is output with white incident light of 52 μW cm^{-1} at 0 V_G. Violate region is TRPR measurement region. b) is overall light on-off behavior as a function of different light incident power at 1 V_D. c) is magnified image for time-resolved rising and decay analysis.

Supporting section 13: The perspective of junction capacitance

The junction capacitance determines the driving speed in the van der Waals heterojunction device. The junction capacitor is formed by the field effect generated in the space charge region (SCR) around the depletion layer generated in the van der Waals heterojunction. We can express the junction capacitor with the following formula.[12]

$$C_j = \left|\frac{dQ}{dV_a}\right|$$

Where dQ is the difference value of charge across SCR, and V_a is applied voltage bias at source-drain electrodes. This value is determined by the thickness of the depletion layer and the externally applied voltage. From the viewpoint of the charge concentration deeply related to the depletion layer formation, the junction capacitor is expressed by the following equation.[12]

$$C_j = A\sqrt{\frac{q\varepsilon n_e n_p}{2(n_e + n_p)V_j}}$$

Then, the conversion into depletion layer thickness can be summarized by the following formula.[]

$$C_j = \frac{\varepsilon A}{w}$$

Assuming that the characteristics of the van der Waals heterojunction structure is a comprehensive RC substitution circuit, the response speed can be approximated by an RC time constant (τ).[18] The TRPR following Figure S12.1 and S12.2 shows the analysis at the p-n junction and the n-n junction controlled by external voltage application and gate. Figure S12.2 adjusted by external voltage shows the difference in rising photocurrent tendency, which is one-step photocurrent rising (-1 V_D) and two-step photocurrent rising (0 V_D). The thickness of the depletion layer at 0 V_D is smaller than the depletion layer thickness at the reverse of -1V_D, and thus shows the difference in observed different photocurrent rising tendencies.

Furthermore, the rising tendency of the photocurrent at the n-n junction at 1 V_D shows a different phenomenon from the plane secondary photocurrent rise as observed at 0 V_D. Assuming that a very narrow depletion layer has been formed as described in the main menu script with the Schottcky junction of the n-n junction, the consistency of our TRPR results is fully understood. Considering that the TRPR of the 2D material vertical heterojunction reported previously is at the picosecond level, observations at the level of microseconds in our lateral three-stage heterojunction device show that the distance (L) required to separate and harvest the excitons is also a very important factor. More precise analysis of this is needed.

References

[1] O. Lopez-Sanchez, D. Lembke, M. Kayci, A. Radenovic, A. Kis, *Nat. Nanotechnol.* **2013**, *8*, 497.

[2] D. Kufer, T. Lasanta, M. Bernechea, F. H. L. Koppens, G. Konstantatos, *ACS Photon.* **2016**, *3*, 1324.

[3] C. H. Lee, G. H. Lee, A. M. van der Zande, W. Chen, Y. Li, M. Han, X. Cui, G. Arefe, C. Nuckolls, T. F. Heinz, J. Guo, J. Hone, P. Kim, *Nat. Nanotechnol.* **2014**, *9*, 676.

[4] H. S. Ra, A. Y. Lee, D. H. Kwak, M. H. Jeong, J. S. Lee, ACS Appl. Mater. Inter. **2018**, *10*, 925.

[5] M. Yamamoto, S. Nakaharai, K. Ueno, K. Tsukagoshi, *Nano Lett.* **2016**, *16,* 2720.

[6] P. Zhao, D. Kiriya, A. Azcatl, C. Zhang, M. Tosun, Y.-S. Liu, M. Hettick, J. S. Kang, S. McDonnell, S. KC, J. Guo, K. Cho, R.Wallace, A. Javey, *ACS Nano* **2014**, *8*, 10808.

[7] Y. Cao, V. Fatemi, S. Fang, K. Watanabe, T. Taniguchi, E. Kaxiras, P. Jarillo-Herrero, *Nature* **2018**, *556*, 43.

[8] V. Fatemi, S. Wu, Y. Cao, L. Bretheau, Q. D. Gibson, K. Watanabe, T. Taniguchi, R. J. Cava, P. Jarillo-Herrero, *Science* **2018**, *362*, 926.

[9] K. Chen, X. Wan, J. Wen, W. Xie, Z. Kang, X. Zeng, H. Chen, and J. Xu, *ACS Nano* **2015**, *9*, 10, 9868 - 9876

[10] B. Zheng, C. Ma, D. Li, J. Lan, Z. Zhang, X. Sun, W. Zheng, T. Yang, C. Zhu, G. Ouyang, G. Xu, X. Zhu, X. Wang, A. Pan, *J. Am. Chem. Soc.* **2018**, *140*, 11193 - 11197

[11] M. Tosun, D. Fu, S. B. Desai1, C. Ko, J. S. Kang, D. Lien, M. Najmzadeh, S. Tongay, J. Wu, A. Javey, *Sci. Rep*. **2015**, *5*, 10990

[12] B. L. Anderson;, R. L. Andersn, Hantee Media **2005**.

[13] Sutherland, B. R. et al. *ACS Photon.* **2015**, *2*, 1117.

[14] Kufer, D. Konstantatos, G. *ACS Photon.* **2016**, *3*, 2197.

[15] V. K. Sangwan, H. N. Arnold, D. Jariwala, T. J. Marks, L. J. Lauhon, M. C. Hersam, *Nano Lett.* **2013**, *13,* 4351.

[16] Kufer, D., Lasanta, T., Bernechea, M., Koppens, F. H. L. & Konstantatos, G. *ACS Photon.* **2016**, *3*, 1324.

[17] H. Li, L. Ye, J. Xu, *ACS Photon.* **2017**, *4*, 823.

[18] M. Massicotte, P. Schmidt, F. Vialla, K. G. Schadler, A. Reserbat-Plantey, K. Watanabe, T. Taniguchi, K. J. Tielrooij, F. H. Koppens, *Nat. Nanoftechnol.* **2016**, *11*, 42.
